# Assessing the inter-observer variability of Computer-Aided Nodule Assessment and Risk Yield (CANARY) to characterize lung adenocarcinomas

**DOI:** 10.1371/journal.pone.0198118

**Published:** 2018-06-01

**Authors:** Erica C. Nakajima, Michael P. Frankland, Tucker F. Johnson, Sanja L. Antic, Heidi Chen, Sheau-Chiann Chen, Ronald A. Karwoski, Ronald Walker, Bennett A. Landman, Ryan D. Clay, Brian J. Bartholmai, Srinivasan Rajagopalan, Tobias Peikert, Pierre P. Massion, Fabien Maldonado

**Affiliations:** 1 Department of Internal Medicine, Vanderbilt University Medical Center, Nashville, Tennessee, United States of America; 2 Vanderbilt University, Nashville, Tennessee, United States of America; 3 Department of Radiology, Mayo Clinic, Rochester, Minnesota, United States of America; 4 Department of Internal Medicine, Division of Allergy, Pulmonary and Critical Care, Vanderbilt University Medical Center, Nashville, Tennessee, United States of America; 5 Veterans Affairs Tennessee Valley Health Care System, Nashville, Tennessee, United States of America; 6 Center for Quantitative Sciences, Vanderbilt University Medical Center, Nashville, Tennessee, United States of America; 7 Department of Physiology and Biomedical Engineering, Mayo Clinic, Rochester, Minnesota, United States of America; 8 Department of Radiology, Vanderbilt University Medical Center, Nashville, Tennessee, United States of America; 9 Department of Electrical Engineering, Vanderbilt University, Nashville, Tennessee, United States of America; 10 Institute of Image Science, Vanderbilt University Medical Center, Nashville, Tennessee, United States of America; 11 Division of Pulmonary and Critical Care Medicine, Mayo Clinic, Rochester, Minnesota, United States of America; Institute of Automation Chinese Academy of Sciences, CHINA

## Abstract

Lung adenocarcinoma (ADC), the most common lung cancer type, is recognized increasingly as a disease spectrum. To guide individualized patient care, a non-invasive means of distinguishing indolent from aggressive ADC subtypes is needed urgently. Computer-Aided Nodule Assessment and Risk Yield (CANARY) is a novel computed tomography (CT) tool that characterizes early ADCs by detecting nine distinct CT voxel classes, representing a spectrum of lepidic to invasive growth, within an ADC. CANARY characterization has been shown to correlate with ADC histology and patient outcomes. This study evaluated the inter-observer variability of CANARY analysis. Three novice observers segmented and analyzed independently 95 biopsy-confirmed lung ADCs from Vanderbilt University Medical Center/Nashville Veterans Administration Tennessee Valley Healthcare system (VUMC/TVHS) and the Mayo Clinic (Mayo). Inter-observer variability was measured using intra-class correlation coefficient (ICC). The average ICC for all CANARY classes was 0.828 (95% CI 0.76, 0.895) for the VUMC/TVHS cohort, and 0.852 (95% CI 0.804, 0.901) for the Mayo cohort. The most invasive voxel classes had the highest ICC values. To determine whether nodule size influenced inter-observer variability, an additional cohort of 49 sub-centimeter nodules from Mayo were also segmented by three observers, with similar ICC results. Our study demonstrates that CANARY ADC classification between novice CANARY users has an acceptably low degree of variability, and supports the further development of CANARY for clinical application.

## Introduction

Now that screening for lung cancer is nationally recommended in most guidelines, the incidence of early lung cancer detection is likely to rise [1]. While this offers a remarkable opportunity to intervene early in the disease course, individualized management of lung cancer therapy will require appropriate risk stratification. Given our evolving knowledge of lung cancer, and the increasingly frequent radiologic detection of tumors that are more indolent than their clinically detected counterparts, over-diagnosis and over-treatment of clinically inconsequential disease are considerable problems. An estimated 20% of cancers diagnosed during the National Lung Screening Trial (NLST) were felt to be slow growing and clinically insignificant, and nearly all of those cancers belonged to the adenocarcinoma (ADC) classification [2–4]. Lung ADC is increasingly recognized as a disease spectrum with varying degrees of aggressiveness, ranging from minimally invasive adenocarcinoma (MIA) and adenocarcinoma in situ (AIS) with nearly 100% post-resection survival to invasive adenocarcinoma (IA) that behaves similarly to other non-small cell lung cancers [5]. Comprehensive semi-quantitative histologic assessment of resected ADCs correlates well with patient outcomes, but cannot by definition be used to guide non-invasive management. Non-invasive characterization of lung ADCs using CT-based quantitative tools could be useful to individualize treatment of lung ADCs.

Computer-Aided Nodule Assessment and Risk Yield (CANARY) is a novel computer software that provides early ADC risk stratification based upon defined radiologic characteristics, which correlate well with known histopathologic features [6,7]. The software detects nine distinct classes of nodule characteristics based on voxel histogram features within CT images of pulmonary ADCs. These features are color coded as Violet (V), Indigo (I), Blue (B), Green (G), Yellow (Y), Orange (O), Red (R), Cyan (C), and Pink (P). The V, I, R, and O (VIRO) classes represent more solid density voxels and are strongly associated with histologic invasion. The B, C, and G classes represent ground-glass density and a spectrum of lepidic growth. Classes P and Y represent voxels categorized between lepidic and invasive growth. The relative presence of these voxel classes within an ADC has been shown to correlate well with post-resection survival, suggesting that non-invasive CANARY assessment could serve as a surrogate for the histologic examination of the tumor.

While CANARY utilizes semi-automatic segmentation based on seed-voxel growing features to isolate an ADC from normal lung parenchyma, software users must verify and manually adjust the borders, particularly if normal solid tissue, such as large vessels, chest wall or mediastinal structures are included in the segmentation. As manual border selection could vary substantially from one software user to the next, the segmentation differences could introduce enough variability to alter CANARY analysis and ADC characterization. Therefore, the inter-observer variability of CANARY must be studied before it can be used clinically. To determine the inter-observer variability of CANARY ADC characterization, three novice software users from two different institutions segmented 95 primary lung ADCs independently and compared outcomes of CANARY characterization. To determine whether smaller nodules were more susceptible to inter-observer variability, segmentations of 49 sub-centimeter nodules from Mayo were also evaluated.

## Materials and methods

### Study subjects

All cases were provided retrospectively from ongoing IRB-approved studies at Vanderbilt University Medical Center (VUMC), Nashville Veterans Administration Tennessee Valley Healthcare System (TVHS), and the Mayo Clinic (Mayo). This study was approved by the institutional review boards at both institutions (IRB numbers 000616 for VUMC, 030763 for TVHS, and 14–000666 for Mayo Clinic, Rochester, MN). The Mayo IRB waived the requirement for informed consent. VUMC/TVHS patients provided written consent to have data from their medical records used for research purposes. Inclusion criteria were patients over age 18 with lung nodule size between 4 and 30 mm identified by low dose chest computed tomography (CT) scan and lung ADC confirmed on pathology from biopsies or surgical resections. Imaging studies were exchanged via institutional computer servers. All imaging studies and clinical data were de-identified before the any of the study researchers accessed the data. Table 1 shows the demographics and tumor staging for the cohort examined in this study.

**Table 1:** Patient demographics, smoking status, and tumor stage.

Demographics	VUMC/TVHS (n = 50)	Mayo (n = 45)
Mean age at diagnosis	67	68.6
Male Gender, n (%)	37 (74)	18 (40)
Smoker, n (%)		
Current	15 (30)	8 (17)
Former	31 (62)	36 (80)
Never	4 (8)	6 (13)
TNM stage, n (%)	26 (52)	32 (71)
IA	8 (16)	5 (11)
IB	2 (4)	1 (2)
IIA	2 (4)	2 (4)
IIB	1 (2)	4 (9)
IIIB	1(2)	1 (2)
IV	0	0

### CT image acquisition

For the 50 patient cohort who had CT scans performed at VUMC or TVHS, scans were performed between 2009 and 2015. All of the lung nodules were confirmed to be ADC after surgical resection. ADCs greater than 3cm were excluded from this study. CT reconstruction algorithms varied by scanner brand/model and included both smooth (soft tissue) and high-resolution (lung) filtered back-projection algorithms. The TVHS CT scanner was a 64-slice helical VCT from GE Medical Systems (Waukesha, WI, USA). The VUMC CT scanner was an 8-slice helical STE scanner from GE Medical Systems (Waukesha, WI, USA). CT Imaging from both scanners was acquired at 120 KVP, with automatic adjustment of milliampere-seconds (range 30–400) to minimize radiation dose. Images on these scanners were reconstructed using a 512-matrix with slice thickness 1.25–2.5 mm and field of view adjusted for each patient to include the entirety of the chest wall and both lungs.

All patients from Mayo had CT scans performed between 2009 and 2015. The Mayo CT images were obtained through a variety of scanners: including GE Medical Systems LightSpeed Ultra, LightSpeed VCT, or LightSpeed Pro 16 (Waukesha, WI, USA); Toshiba Aquilion (Tustin, CA); Siemens Sensation 16 and Sensation 64 (Malvern, PA). CT reconstruction algorithms varied by scanner brand/model and included both smooth (soft tissue) and high-resolution (lung) filtered back-projection algorithms. Images on these scanners were reconstructed using a 512-matrix with slice thickness 1.25–2.5 mm and field of view adjusted for each patient to include the entirety of the chest wall and both lungs.

### CANARY observers

Authors ECN, MPF, and TFJ served as the three observers who evaluated CT images of ADCs and performed CANARY analysis upon the cohort of 50 patients from VUMC/TVHS and 45 patients from Mayo. ECN was a second-year Internal Medicine resident at VUMC, and she had received introductory radiology training as a medical student. MPF was a fourth-year undergraduate student at Vanderbilt University, and had no prior radiology training. TFJ was a thoracic radiology fellow in the sixth year of his radiology training. SR, BJB, and RK segmented independently an additional cohort of 49 nodules from Mayo that were less than 1 centimeter. All three of these authors have extensive experience in thoracic radiology.

### Nodule definition, localization and segmentation

The location of each ADC was confirmed by one radiologist (TFJ) and one pulmonologist (FM), and a screenshot of the nodule was shared amongst the three software users (TFJ, ECN, MPF). Two observers at VUMC/TVHS and two of the CANARY developers at Mayo established a user standard operating procedure (SOP, S1 File) as they learned to use the software. The SOP was then shared with the third observer at Mayo. The SOP provided instructions for consistent ADC identification in all three CT views (axial, coronal, and sagittal), use of image zooming features, and most importantly for adjustment of the ADC border. When a nodule abutted the chest wall, mediastinal structures, or large blood vessel, the observer was instructed to draw an exclusionary line or use a “nudge” tool to exclude this tissue as the observer saw fit. If the observer judged that CANARY’s automated segmentation involved the nodule alone, the automated nodule borders were not adjusted.

**Fig 1:**
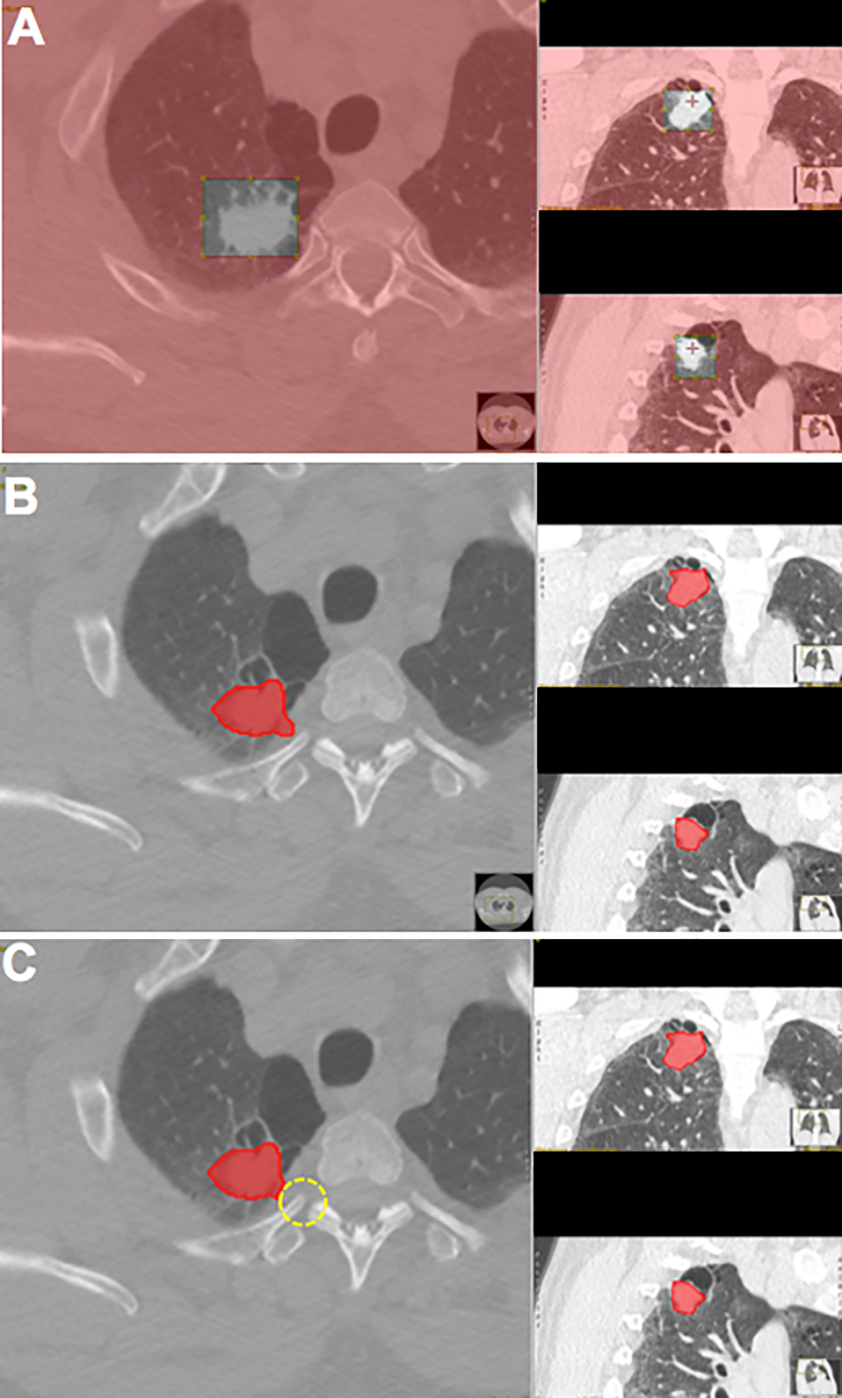
CANARY segmentation process. CT scans from a cohort of biopsy-confirmed ADCs from VUMC/TVHS (n = 50) and Mayo (n = 45) were analyzed by three independent software users from two institutions using CANARY segmentation. All ADCs were <3 cm, according to previous CANARY studies. A pulmonologist and radiologist confirmed nodule location prior to segmentation. **A.** Once the observer had identified the nodule on the CT scan, placing the pointer over the nodule established a volume of interest around the nodule. **B.** Next, CANARY defined the nodule border (red area) on each CT slice. **C.** If the border appeared inconsistent with the perceived nodule edge (i.e. extension into vasculature or chest wall), the observer adjusted the nodule borders by using an eraser tool (dotted yellow circle).

Five randomly selected ADCs from the VUMC/TVHS cohort were segmented by the three observers according to the SOP. Segmentation data from these five ADCs was shared amongst the observers to confirm similar results of segmentation according to the SOP prior to further segmentation of the entire study cohort. Data from these five ADCs was included in the final study. The software users were blinded to patient outcomes prior to segmentation. Fig 1 illustrates the process of segmentation with an example case.

### CANARY analysis

Once the observer confirmed or adjusted the ADC borders, CANARY classification of the ADC was performed. This method has been published previously [6,7]. Briefly, CANARY analysis was developed using machine learning techniques based upon the analysis of 37 histologically proven lung ADCs that had pre-surgical, volumetric, non-contrast high-resolution CT data obtained for clinical reasons. These nodules had various degrees of histologic invasion, and CT characteristics spanning the spectrum of pure groundglass opacity through fully solid density. 774 volumes of interest (VOI) of 9x9 voxels within the 37 nodules were arbitrarily selected, and histogram/texture characteristics of these VOIs were compared using pairwise similarity metrics. Affinity propagation, an unsupervised clustering algorithm, and pairwise similarity metrics identified radiologically similar VOI clusters and the centroid of these clusters was established as the phenotype of each cluster/characteristic. These classes were color coded as Violet (V), Indigo (I), Blue (B), Green (G), Yellow (Y), Orange (O), Red (R), Cyan (C), and Pink (P). The imaging classifications represented a spectrum of histologic features of malignancy from predominantly lepidic growth to invasive ADC.

**Fig 2:**
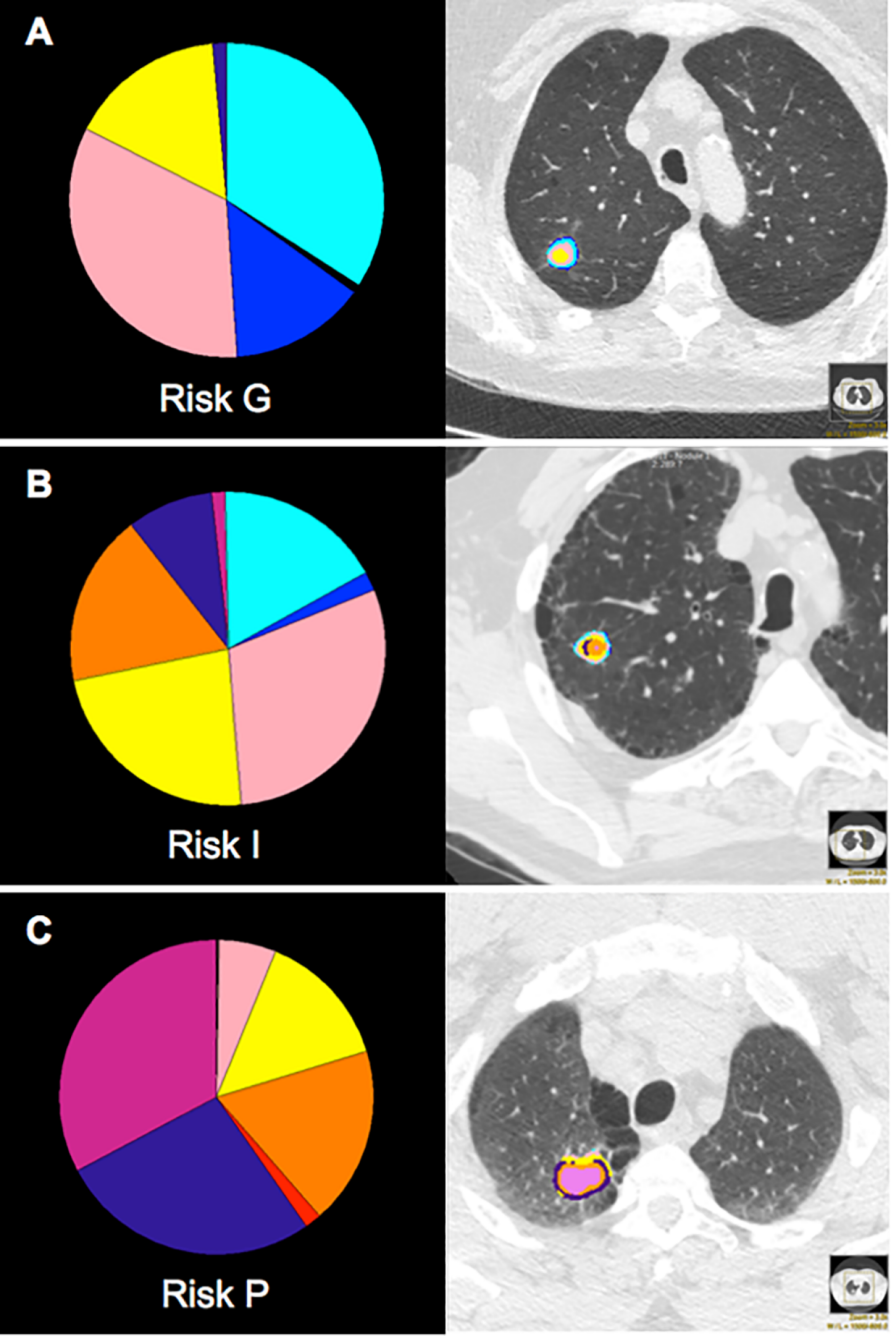
ADC characterization based upon the CANARY class composition. CANARY detected nine unique voxel characteristics within CT image data, which were color coded as CANARY classes: Violet (V), Indigo (I), Blue (B), Green (G), Yellow (Y), Orange (O), Red (R), Cyan (C), and Pink (P). Voxels of class V, I, R, and O (VIRO) were associated with invasion, while the classes B, C, and G represented lepidic growth. P and Y class voxels were between lepidic and frankly invasive growth, such those found in MIA or AIS. The composition of each class within the total ADC voxels was used to define overall ADC risk characterization as Good (G), Intermediate (I), or Poor (P). Above are three ADCs at the completion of CANARY analysis and characterization. **A**, **B**, and **C** are examples of G, I, and P nodule characterizations respectively.

CANARY analysis of a nodule was performed by assessing each voxel in the nodule segmentation with its surrounding 80 voxels (a 9 x 9 voxel) sequentially. The histogram features of this voxel were classified as one of the nine defined classes based on similarity. The relative representation of each voxel class within the entire nodule establishes the parametric signature of the entire nodule, which has been shown to correlate with both histology and patient outcomes. Fig 2 shows the CANARY voxel class composition within an ADC as well as the overall prognostic characterization (Good (G), Intermediate (I), and Poor (P)) established by the ADC composition. This prognostic characterization was established in previous CANARY publications, and relates to overall postoperative survival.

### Statistical analysis

The primary goal of this study was to assess the variability in CANARY analysis between three different observers. The detection of the individual nine CANARY classes and the cumulative composition of a whole ADC were compared between observers’ segmentations. The variance of each CANARY class was calculated to assess the deviation of each observer’s segmentation from the mean of the three observers’ segmentation. Intraclass Correlation Coefficient (ICC) is the ratio of the between-nodule variance to the total variance, where the total variance is the sum of between-nodule variance, between-observer variance and unexplained variation. As between-nodule variance increases, the relative contribution of between-observer variance to the total variance decreases. Using a linear mixed effect model, the ICC was calculated to evaluate reproducibility of each voxel class detected within CT images of ADCs. Additionally, the Dice similarity coefficient (DSC) was calculated to compare the similarity of segmentations between any two observers for each ADC examined. Kruskal-Wallis test was performed to determine whether there was a significant difference in ICC results when CT scans were categorized by slice thickness. Fleiss kappa coefficient was used to assess the agreement of CANARY prognostic characterizations for ADCs among three observers. Statistical calculations were performed in R 3.3.2 (R Foundation for Statistical Computing; Vienna, Austria).

## Results

### Inter-observer agreement of classes detected in CANARY segmentation

For each segmentation, CANARY provided a tally of segmented voxels categorized under each class and the total segmented voxel count. The percentage of each class within an ADC established the ADC’s prognostic characterization. Therefore, differences in the relative composition of an ADC based upon an observer’s segmentation could have significant clinical implications for a given lesion.

**Fig 3:**
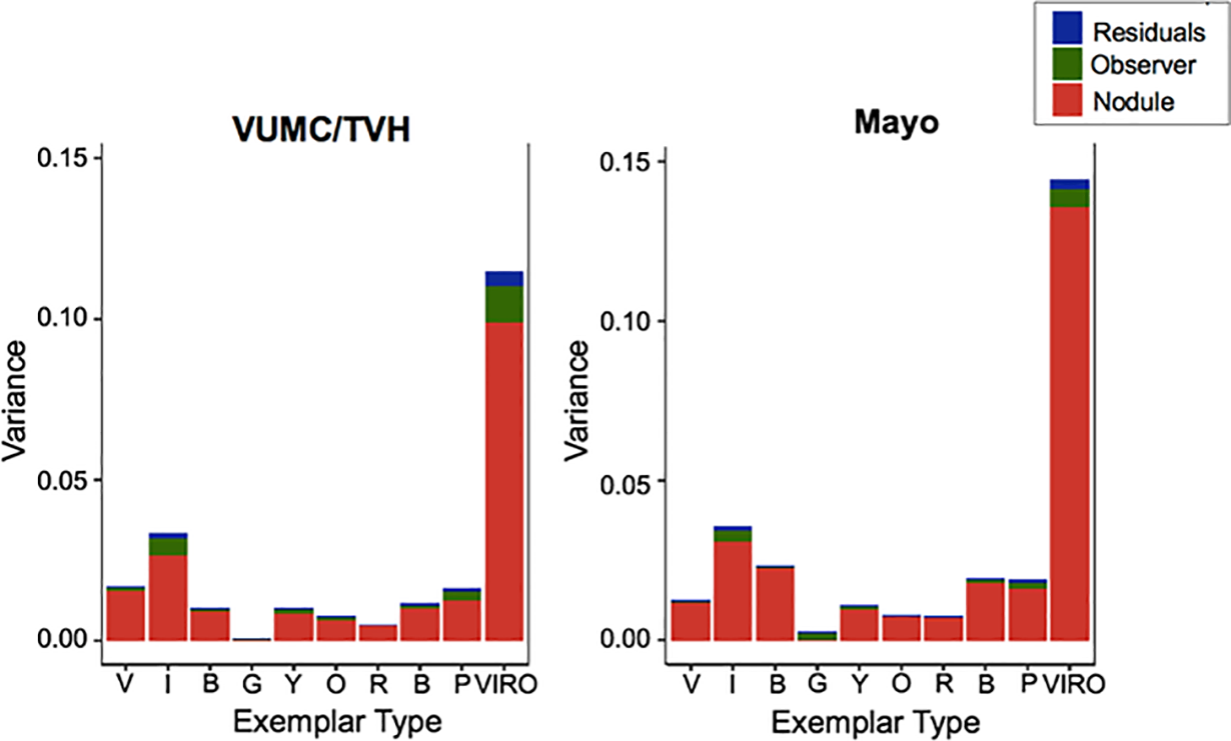
Observer segmentation differences contribute minimally to the variance of CANARY classes. The variance between observer segmentations was calculated for each CANARY class and the V, I, R, O (VIRO) classes collectively. CANARY classification variance was compared between the individual ADCs (red), inter-observer segmentations (green), or residual intrinsic variability (blue).

Variance analysis was performed to assess how the observers’ segmentations, the ADCs, or intrinsic variability contributed to differences in the percentage of each voxel class within an ADC. Variance was calculated for each class as well as for classes V, I, R, and O (VIRO) collectively, which represent the most invasive nodule features identified on CT. Fig 3 shows the contributions of the observer, the ADC nodule, and intrinsic residual variability to the variance of each CANARY voxel class. Differences in the ADCs’ voxel class composition generated the greatest source of variance (between-nodule variance), while the individual observer segmentations contributed minimally to variance.

Next, to assess the reproducibility of CANARY class voxel counts between observer segmentations, the ICC was calculated for each class and VIRO based upon the percentage of each class within the entire ADC. Table 2 shows the ICC values for individual CANARY classes, the VIRO group, and the average of all classes. The averaged ICC for all of the classes was 0.828 (95% CI 0.76, 0.895) for the VUMC/TVHS cohort, and 0.852 (95% CI 0.804, 0.901) for the Mayo cohort. The G class had the lowest ICC score of the nine classes.

**Table 2:** Intra-class correlation coefficient (ICC) is highest amongst the CANARY classes representing most invasive ADC features. Intra-class correlation coefficient (ICC), a measure of agreement of quantitative assessments made by different observers evaluating the same quantity, was calculated for each CANARY class, the VIRO group, and the average (Avg.) of all classes. An ICC of 0.8–1 reflects high agreement between users. These calculations were performed for the VUMC/TVHS and the Mayo cohorts, as well as for each subgroup of patients scanned by a particular CT scanner (GE Medical Systems, Philips, and Siemens). ICCs and 95% CI for each voxel class are shown below. The number of patients within each cohort is listed in parentheses.

	Avg.	V	I	B	G	Y	O	R	C	P	VIRO
**All cases**											
VUMC/TVHS	0.828	0.925	0.795	0.91	0.467	0.827	0.864	0.977	0.868	0.778	0.865
(50)	(0.76, 0.895)	(0.891, 0.959)	(0.71, 0.88)	(0.87, 0.951)	(0.302, 0.632)	(0.754, 0.901)	(0.804, 0.923)	(0.966, 0.988)	(0.81, 0.926)	(0.687, 0.869)	(0.805, 0.924)
Mayo	0.852	0.953	0.871	0.963	0.151	0.881	0.965	0.99	0.939	0.866	0.942
(45)	(0.804, 0.901)	(0.93, 0.976)	(0.811, 0.931)	(0.945, 0.981)	(-0.035, 0.338)	(0.826, 0.937)	(0.947, 0.982)	(0.985, 0.995)	(0.909, 0.969)	(0.804, 0.928)	(0.913, 0.97)
**GE Medical Systems**											
VUMC/TVHS	0.737	0.923	0.736	0.492	0.275	0.822	0.906	0.872	0.646	0.863	0.835
(25)	(0.608, 0.866)	(0.874, 0.973)	(0.588, 0.884)	(0.264, 0.72)	(0.275, 0.529)	(0.715, 0.928)	(0.847, 0.966)	(0.793, 0.952)	(0.463, 0.83)	(0.779, 0.948)	(0.735, 0.935)
Mayo	0.851	0.939	0.833	0.991	0.195	0.858	0.972	0.983	0.951	0.857	0.933
(22)	(0.778, 0.924)	(0.897, 0.981)	(0.725, 0.94)	(0.984, 0.997)	(-0.075, 0.465)	(0.764, 0.951)	(0.952, 0.992)	(0.97, 0.995)	(0.916, 0.985)	(0.763, 0.951)	(0.887, 0.979)
**Philips**											
VUMC/TVHS (24)	0.831	0.929	0.829	0.921	0.464	0.841	0.798	0.976	0.92	0.747	0.883
	(0.736, 0.926)	(0.882, 0.976)	(0.724, 0.934)	(0.869, 0.973)	(0.226, 0.703)	(0.743, 0.94)	(0.677, 0.919)	(0.96, 0.992)	(0.868, 0.973)	(0.601, 0.893)	(0.809, 0.958)
**Siemens**											
Mayo (21)	0.853	0.966	0.903	0.942	0.124	0.9	0.958	0.999	0.93	0.862	0.945
	(0.784, 0.921)	(0.941, 0.991)	(0.837, 0.97)	(0.901, 0.983)	(-0.146, 0.394)	(0.83, 0.969)	(0.927, 0.988)	(0.999, 1)	(0.881, 0.979)	(0.763, 0.955)	(0.905, 0.984)

Given the potential impact of different CT scanner manufacturer settings, the cohort data was separated according to the CT scanner manufacturer, and the ICCs for each CANARY class, the average, and VIRO group were calculated (Table 2). Two patients from Mayo and one patient from VUMC/TVHS were scanned on Toshiba scanners, and separate ICC calculations were not performed for this small cohort. The ICC for each of the three CT manufacturer subgroups revealed the same pattern as for the cohort as a whole: the ICC was consistently >0.9 in the VIRO group, and >0.7 in the average of all CANARY classes. Voxel class G had the lowest ICC in all manufacturer subgroups.

The impact of different CT slice thicknesses upon observers’ segmentations was also considered. The CT scans were sorted by slice thickness, ranging from 1.0 to 2.5mm, and the ICCs for each CANARY voxel class were calculated (S1 Table). Five of the 95 CT scans from Mayo and VUMC/TVHS, obtained at 3mm (n = 3) and 5mm (n = 2) thickness, were excluded from analysis due to low samples size that those thicknesses. Kruskal-Wallis test revealed that there was not a significant difference in ICCs when the CT scans were categorized by slice thickness (H = 2.421, p = 0.659).

**Fig 4:**
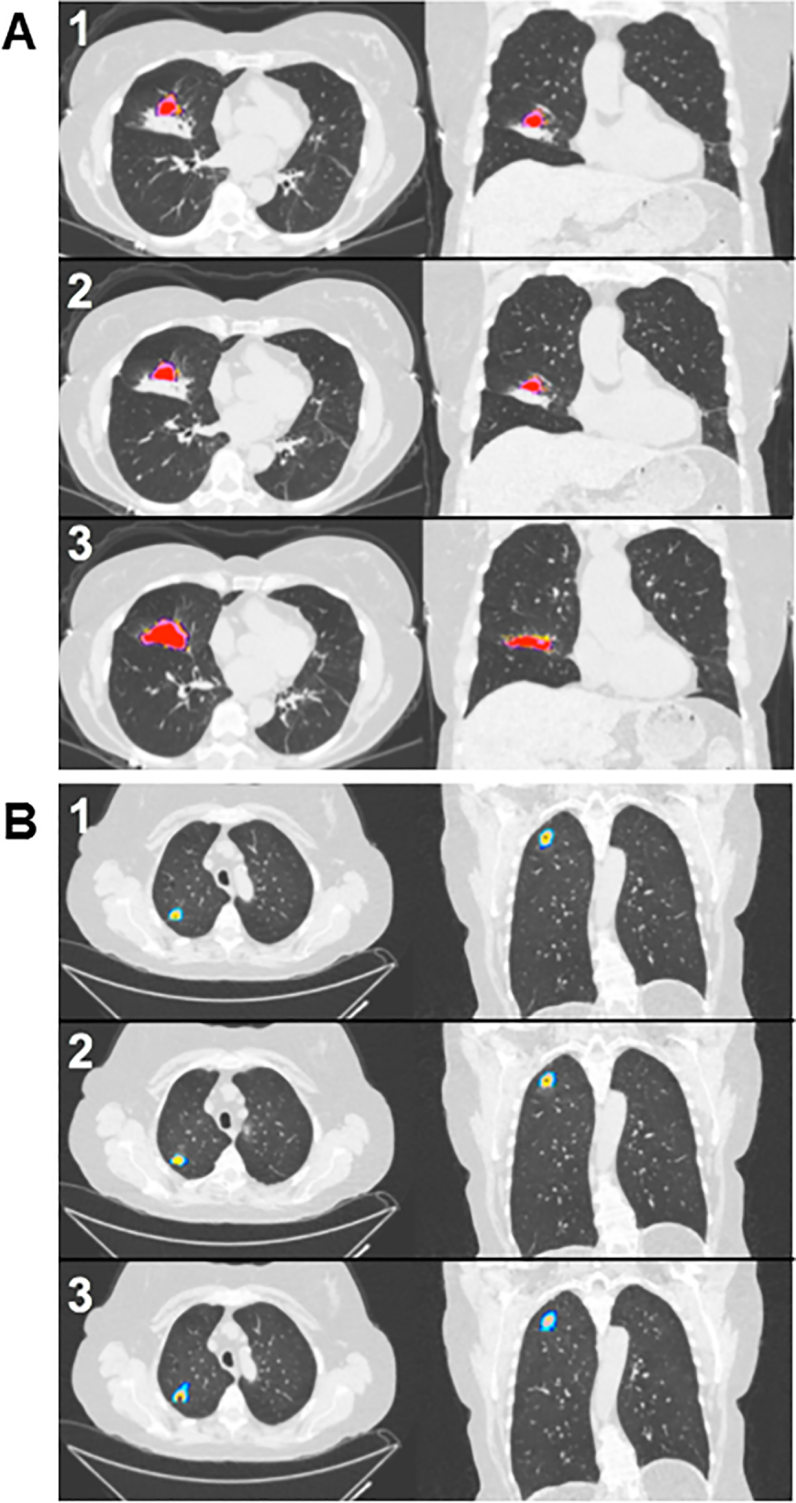
CANARY class patterns between observers. Two ADC cases are shown with the segmentations as completed by the three observers. The segmentations numbered 1 and 2 for each case were performed by a VUMC/TVH observer, while segmentation numbered 3 was performed by the Mayo observer. **A.** The VUMC/TVHS observers and the Mayo observer segmented different portions of this nodule while following the standard operating procedure (SOP), yet this had minimal impact upon the nodule class composition as shown by the high percentage of the R class in the nodule. **B.** A representative case showing low segmentation variability between observers.

DSC values, which assessed the segmentation uniformity between observers, further demonstrated inter-observer consistency of CANARY analysis. Four of the VUMC/TVH cases did not generate the proper files required for DSC calculation after they were segmented in CANARY, and these were excluded from statistical analysis. The mean DSC for the 46 VUMC/TVH cases was 0.793 (standard deviation 0.125, 95% CI 0.772, 0.814); for the 45 Mayo cases, the mean DSC was 0.812 (standard deviation 0.103, 95% CI 0.795, 0.829). DSC > 0.7 indicates strong overlap and is used in segmentation validation literature as a threshold for acceptable reproducibility [8]. Fig 4 compares CANARY ADC characterization assignment based upon the three observer segmentations of the same case as an example.

To determine whether smaller nodule size influenced inter-user variability, an additional cohort of 49 nodules from Mayo, all less than 1cm in diameter, was segmented and ICCs calculated for the voxel classes (Table 3). Again, the ICC of the voxel class average was greater than 0.8, and the VIRO group had the highest ICC of all classes.

**Table 3:** Intra-class correlation coefficient (ICC) amongst CANARY voxel subtypes from nodules less than 1cm in diameter. Avg. is the average of all voxel classes. 95% CI is shown in parentheses below the ICC.

	Avg.	V	I	B	G	Y	O	R	C	P	VIRO
Mayo (49)	0.849	0.93	0.855	0.87	0.584	0.788	0.942	0.98	0.85	0.783	0.907
	(0.787, 0.911)	(0.898, 0.962)	(0.792, 0.919)	(0.813, 0.928)	(0.438, 0.729)	(0.7, 0.877)	(0.915, 0.969)	(0.97, 0.989)	(0.785, 0.916)	(0.693, 0.873)	(0.864, 0.949)

### Significant inter-observer agreement of CANARY risk stratification

Based upon an ADC’s relative composition of the nine classes, CANARY generates a prognostic characterization of good (G), intermediate (I), or poor (P) based upon Kaplan-Meier survival curves from initial CANARY studies [9], as shown in Fig 2. Fleiss Kappa, a measure of chance-adjusted measure was applied to the prognostic characterizations generated by the observers’ segmentations. A Kappa score of 0.61–0.8 represents substantial agreement, and a score of 0.81–1.0 represents perfect agreement. For the VUMC/TVHS cohort, the Kappa score was 0.75 (95% CI 0.62, 0.88); for the Mayo cohort, the kappa score was 0.82 (95% CI 0.7, 0.94).

## Discussion

By characterizing the distributions of nine distinct radiologic voxel classes within ADC nodules, CANARY analysis has been shown to correlate well with histology and patient outcomes in previous studies [6,9], and could prove useful for the objective analysis of these radiologic opacities. Our study demonstrated low inter-observer variability with nodule analysis performed by three novice CANARY users, indicating that the CANARY results published previously could be generalized to users with limited software experience, and establish CANARY as a valuable tool to characterize early lung ADCs.

The collective VIRO classes had the highest ICC amongst all classes in both institutional cohorts. VIRO are detected in invasive ADCs based upon histologic comparison. Our findings indicate that CANARY can identify more aggressive ADCs consistently when nodules are segmented by different individuals. For CANARY classes B and C, which represent lepidic growth patterns, as well as for classes P and Y, which correlate to tissue that is between invasive and lepidic growth, ICC values also met criteria for acceptably high reproducibility. The influence of manually adjusting ADC borders therefore had minimal impact overall upon CANARY analysis in a diversity of ADC phenotypes.

The G class, representing the most lepidic features on CT, had the lowest ICC value in both institutional cohorts. There are two potential explanations for this low reproducibility between observer’s segmentations. First, few of the ADCs in our cohorts contained a significant percentage of G class voxels, magnifying any relative difference in the measurement of G voxels in our cohort. 89% of ADC segmentations from all three observers had <5% of their voxels classified as G. This was a limitation of our study. Secondly, the low reproducibility of the G class quantification between users may also be a consequence of the challenges in defining lepidic tissue borders, which are found in ground glass opacities (GGOs). Proper border delineation of pulmonary lesions containing lepidic growth may require greater radiology expertise to achieve low inter-user variability, however it is acknowledged amongst experienced radiologists that establishing borders for these lesions is challenging. There are no universal guidelines at present for defining GGO borders [10], which can represent the AIS and MIA subclasses of ADCs. Assessing CANARY inter-observer variability in a retrospective analysis of AIS and MIA nodules that tend to contain G class voxels could refine our SOP. Ultimately, these discrepancies may in fact have minimal clinical implications given that the distribution of CANARY classes representing histologic invasion appear to have the greatest impact upon ADC prognosis, and the detection of these classes had low variability and high reproducibility between users.

Our findings help to advance the application of radiomics in the field of early lung cancer assessment. Radiomics, the extraction and analysis of data from medical images to guide clinical decision making, is being applied to prostate, liver, and breast cancer assessments. Textural analysis of diffusion and T2-weighted magnetic resonance images (MRI) in patients with prostate cancer has been shown to help distinguish cancerous from noncancerous tissues. This method has also been shown to improve differentiation of prostate cancer with Gleason scores of 6, which may only require active surveillance, from malignancies with Gleason scores of 7, which may require more aggressive intervention [11–13]. Kuo et al examined imaging from patients with hepatocellular carcinoma, and identified CT phenotypes that correlated with a doxorubicin drug-response gene expression program, suggesting these imaging phenotypes could guide individualized treatment [14]. Teruel et al applied textural analysis to dynamic contrast enhanced breast MRI, and found that this assessment could provide prediction of a patient’s response prior to receiving neoadjuvant chemotherapy [15].

The accuracy and reliability of radiomics methods when applied to images collected by different machines or by the same machine under slightly different settings has been an area of concern and ongoing investigation within this field [16,17]. It is generally accepted that CT slice thickness for lung nodule evaluation is optimal at 1–2.5mm, and 90- of the total 95 CT scans in our study met that criteria [18]. While greater slice thickness reduces nodule resolution, this metric did not have a significant upon inter-observer agreement of ADC composition by CANARY voxel type. When our cohort data was categorized by CT manufacturer, the ICC values for each scanner type followed the same pattern as the cohort as a whole with the most invasive voxel classes having the highest ICC, and the G class having the lowest ICC. This subgroup analysis demonstrates that within this cohort and amongst these CT scanners, CANARY classifications of ADCs have low inter-observer variability. This finding has important clinical implications as medical centers evaluating patients with early lung ADC use a variety of CT scanners and reconstruction settings. Differences in the radiomics metrics utilized for CANARY ADC classification do not appear to influence inter-observer agreement significantly.

Radiomic applications to risk stratify lung ADCs may play a fundamental role in precision oncologic care for the most common lung malignancy. Current standard of care strategies for early stage lung ADC, if indiscriminately applied, almost certainly result in overtreatment of indolent tumors that patients, particularly those with extensive comorbidities, are more likely to die with than from. The increasing awareness of an aggressiveness spectrum within the ADC landscape is driving interest in alternative therapeutic strategies such as limited resections, stereotactic body radiation therapy, and even active surveillance for the most indolent lesions [19–22]. As other non-invasive methods of ADC characterization are developed, such as additional imaging techniques or liquid biopsy, comparing or combining these methods with CANARY may strengthen diagnostic sensitivity and specificity [23]. On a molecular level, the relationship between tumor biology and radiologic features should be assessed by investigating the presence of protein or genetic markers that correlate with nodule radiology findings. While this research is in its initial phase, non-invasive biomarkers that could facilitate ADC characterization could prove invaluable, and are generating considerable interest among investigators.

Further validations of CANARY as a clinical tool include prospective testing of CANARY analysis to determine whether there is a relationship between CANARY-derived prognostic stratification. This study demonstrates that CANARY analysis is reliable between observers, and serves as an important step in moving CANARY closer to clinical application.

## Supporting information

S1 File Standard operating protocol. This document provided step by step instruction on ADC segmentation using CANARY software. This document provides step-by-step instructions for nodule segmentation using CANARY software. It was developed by authors ECN, MPF, TFJ, RK, and SR. The SOP is also available at protocols.io under the title, “CANARY Segmentation of Lung Adenocarcinoma”, and may be found at dx.doi.org/10.17504/protocols.io.mrcc52w.
(PDF)

S1 Table CT scan slice thickness does not impact intra-class correlation coefficients (ICCs) of CANARY voxel subtypes. S1 Table shows the ICCs for each CANARY voxel type when the CT scans were sorted by slice thickness, ranging from 1.0 to 2.5mm. Five of the 95 CT scans from Mayo and VUMC/TVHS, obtained at 3mm (n = 3) and 5mm (n = 2) thickness, were excluded from analysis due to low samples size that those thicknesses. Kruskal-Wallis test revealed that there was not a significant difference in ICCs when the CT scans were categorized by slice thickness (H = 2.421, p = 0.659).
(DOCX)
