# Supplementary material for: Assessing the inter-observer variability of Computer-Aided Nodule Assessment and Risk Yield (CANARY) to characterize lung adenocarcinomas
Source: PLoS One. 2018 Jun 1;13(6):e0198118. doi: 10.1371/journal.pone.0198118 (PMC5983856; doi:10.1371/journal.pone.0198118)
Supplement: S1 File — This document provided step by step instruction on ADC segmentation using CANARY software. This document provides step-by-step instructions for nodule segmentation using CANARY software. It was developed by authors ECN, MPF, TFJ, RK, and SR. The SOP is also available at protocols.io under the title, “CANARY Segmentation of Lung Adenocarcinoma”, and may be found at dx.doi.org/10.17504/protocols.io.mrcc52w. (PDF) [file pone.0198118.s001.pdf]

CANARY Segmentation of Lung Adenocarcinoma

Available at protocols.io: dx.doi.org/10.17504/protocols.io.mrcc52w

A. Finding the nodule
    1. Scroll up and down in axial view to identify the pulmonary nodule. Scroll through the entirety of the lungs to ensure you are not missing a nodule. (Keep the CT image in "lung view", i.e. Do not change the image view to "soft tissue" if trying to differentiate between solid and sub-solid tissue).
    2. Click the axis tool (defined below) and place it over the nodule so that you can see it in all three views (axial, sagittal, and coronal).
    3. Click the nodule, and a box will be placed around it with the rest of the screen in red.
    4. Ensure the entirety of the nodule is enclosed within the box in all three views. Adjust the box dimensions by clicking and dragging the border to the desired location.

B. Establishing nodule perimeter
    1. If the nodule abuts the pleura or mediastinal structures, exclude these tissues as much as possible from within the box. You can expand the nodule perimeter after it has been established by the software, but it can take a great deal of time to erase tissue that you do not want included in the analysis.
    2. The Wall function may also be used to exclude tissue from the chest wall. Draw a line along the chest wall, clicking once to create distinct points along the line. At the last point, double click to finish the line. Scroll through the CT slices that involve the lung nodule. Adjust the line by moving its distinct points to ensure that the chest wall is appropriately excluded.
    3. Click "Get nodule mask".
    4. A red ROI will appear around the perimeter of the nodule. (Pressing the letter "T" will toggle the mask on and off from the screen, but not permanently remove it.)
    5. Scroll through the slices and adjust the mask with the tools below.
        i. Use the "Nudge" function under the REFINE tab to adjust the perimeter of the nodule.
        ii. Holding down the middle mouse key will enable you to change the size of the Nudge tool.
        iii. If the cursor is within the mask when you begin to nudge, it will EXPAND the mask.
        iv. If the cursor is outside of the mask when you begin to nudge, it will SHRINK the mask.
    6. The Trace function can be used to include tissue of interest into the mask. Draw a line around the tissue, connecting the start and end points of the line to the original mask.
    7. Once finished with all adjustments of ROI, click classify nodule.
    8. Once nodule has been classified for the first time, the "edit nodule" & "delete nodule" buttons can be used to adjust the ROI.

C. Tools

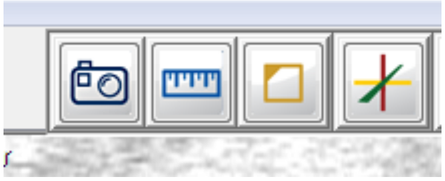

1. Camera tool: Will take a screen shot of the CT scan and import the image to the final report generated for each nodule.
2. Ruler tool: measure structures using cursor.

3. Note tool: Enables you to label structures with a small yellow “sticky note” using the cursor. Right click for options on using the note.
4. Axis tool: Locks axial, sagittal, and coronal views so that you can scroll through the images in a coordinated fashion.

D. Extracting the data
1. Clicking classify will save the data every time in the same Excel file.
2. The Excel data file can be found by opening the C drive → Temp folder: CANARY-Plus folder: “CANARY-PlusTumorStats”.
3. Data from each CT case is listed in a row.
4. Seed, X, Y, Z columns indicate the position of the cursor when the nodule mask was set.
5. Letters V (violet), I (indigo), B (blue), G (green), Y (yellow), O (orange), R (red), C (cyan), and P (pink) represent the 9 colors shown in the “Classify nodule” analysis wheel. The numbers in these columns represent the volume (mm^3) of the nodule that is assigned to each color classification.
6. Ensure that the CANARY-PlusTumorStats file is closed whenever segmentation is being performed. Otherwise, new data will not import into the Excel file.
