# Supplementary material for: Assessing the inter-observer variability of Computer-Aided Nodule Assessment and Risk Yield (CANARY) to characterize lung adenocarcinomas
Source: PLoS One. 2018 Jun 1;13(6):e0198118. doi: 10.1371/journal.pone.0198118 (PMC5983856; doi:10.1371/journal.pone.0198118)
Supplement: S1 Table — shows the ICCs for each CANARY voxel type when the CT scans were sorted by slice thickness, ranging from 1.0 to 2.5mm. Five of the 95 CT scans from Mayo and VUMC/TVHS, obtained at 3mm (n = 3) and 5mm (n = 2) thickness, were excluded from analysis due to low samples size that those thicknesses. Kruskal-Wallis test revealed that there was not a significant difference in ICCs when the CT scans were categorized by slice thickness (H = 2.421, p = 0.659). (DOCX) [file pone.0198118.s002.docx]

S1 Table. **CT scan slice thickness does not impact intra-class correlation coefficients (ICCs) of CANARY voxel subtypes.** Table S1 shows the ICCs for each CANARY voxel type when the CT scans were sorted by slice thickness, ranging from 1.0 to 2.5mm. The number of CT scans grouped in each thickness category is shown in parentheses. The CANARY voxel types are listed in the top row. Five of the 95 CT scans from Mayo and VUMC/TVHS, obtained at 3mm (n = 3) and 5mm (n = 2) thickness, were excluded from analysis due to low samples size that those thicknesses. Kruskal-Wallis test revealed that there was not a significant difference in ICCs when the CT scans were categorized by slice thickness (H = 2.421, p = 0.659).

| **Slice Thickness (mm)**  **(n)** | **V** | **I** | **B** | **G** | **Y** | **O** | **R** | **C** | **P** | **VIRO** |
| --- | --- | --- | --- | --- | --- | --- | --- | --- | --- | --- |
| **1.0**  (16) | 0.956 | 0.923 | 0.908 | 0.676 | 0.886 | 0.932 | 0.975 | 0.935 | 0.886 | 0.963 |
| **1.25**  (42) | 0.917 | 0.820 | 0.975 | 0.484 | 0.820 | 0.957 | 0.983 | 0.940 | 0.842 | 0.906 |
| **1.5**  (17) | 0.980 | 0.933 | 0.933 | 0.430 | 0.886 | 0.973 | 0.982 | 0.927 | 0.853 | 0.960 |
| **2**  (9) | 0.9138 | 0.790 | 0.960 | 0.886 | 0.791 | 0.871 | 0.999 | 0.839 | 0.780 | 0.911 |
| **2.5**  (6) | 0.9613 | 0.859 | 0.085 | 0.973 | 0.912 | 0.905 | 0.986 | 0.727 | 0.960 | 0.912 |
